# Treatment of Aneurism by Compression

**Published:** 1845-12-01

**Authors:** 


*Cure of Aneurism by pressure.*—The attention of surgeons seems to be now directed with considerable interest to the cure of aneurism by pressure, and since every discovery,or improvement which dispenses with the use of the scalpel, is preeminently indicative of surgical progress, it is greatly to be hoped that the expectations of those who advocate this method will be realized. In Ranking's abstract, vol. 1, No. 1, we find several extracts from communications by Dr. Bellingham, of Dublin, originally made to the Dublin Journal on this subject. He gives a statement of 12 cases of popliteal and femoral aneurism permanently cured by compression. Our readers, by consulting the above work, will find a full discussion of the advantages of this method over the treatment by taking up the artery, and a consideration of the objections which have been urged against it. These our limits will not enable us to quote. We will, however, for those who cannot conveniently refer to periodicals which treat at length on the subject, transfer a brief account of the practical measures recommended by Dr. B. In so doing we use the language of Henry Ancell, Esq., author of the report on the progress of surgery, in the work referred to. In addition to Dr. Bellingham, we are informed in this report that the plan has been adopted by Hutton, Cusack, Allen, Greatrex, and Liston. The reporter derives his authority from the late work on surgery by Prof. Miller. "By any suitable apparatus a moderate degree of pressure is applied to the vessel at a point where the coats may be expected to be sound, and, consequently, not prone to ulcerate from various causes. This is maintained so long as it can be conveniently borne by the patient, but no longer. When uneasy sensations become intense, with swelling and numbness of the limb, and throbbing in the part, the pressure is either slackened or removed. After a time, the parts having recovered, it is re-applied. Again it is removed, and thus the disasters formerly attendant upon the treatment by pressure are avoided. At the same time that the circulation in the aneurism is moderated, the tumor begins to diminish, its pulsation is less, and it feels harder and less compressible; ultimately, pulsation wholly disappears, and induration becomes complete, absorption advances, and the cure is obtained, with or without a pervious state of the vessel. Throughout the treatment absolute repose and decumbency are maintained, with an antiphlogistic regimen. Also, the limb below the compressed point must be uniformly and equally supported by bandaging, lest passive congestion and oedema supervene; and this pressure may, from time to time, be somewhat increased on that part of the limb which includes the aneurismal tumour. The process is one of weeks, not days—gradual, not sudden—interrupted—not continuously, progressive. The pressure requires to be neither great nor constant, for we do not desire obliteration, even temporary. It is sufficient to moderate, not essential to obstruct the flow of blood. The treatment is conducted rather as if itself were not the agent of cure, but only the means whereby the spontaneous cure may be originated and favored."

Dr. Bellingham says that "instead of employing a single instrument, we employ two or three, if necessary; these are placed upon the artery leading to the aneurismal sac, and when the pressure of one becomes painful it is relaxed, the other having been previously tightened." "The instrument consists of an arc of steel covered with leather, at one extremity of which is an oblong padded splint; the other extremity terminates in a nut, containing a quick screw, to which a pad similar to that of the tourniquet is attached."

